# Estimating the magnitude and direction of bias in tuberculosis drug resistance surveys conducted only in the public sector: a simulation study

**DOI:** 10.1186/1471-2458-10-355

**Published:** 2010-06-21

**Authors:** Ted Cohen, Bethany L Hedt, Marcello Pagano

**Affiliations:** 1 Division of Global Health Equity, Brigham and Women's Hospital, 641 Huntington Avenue, Boston, 02115, USA; 2 Department of Epidemiology, Harvard School of Public Health, 677 Huntington Avenue, Boston, 02115, USA; 3 Department of Biostatistics, Harvard School of Public Health, 677 Huntington Avenue, Boston, 02115, USA

## Abstract

**Background:**

Accurate assessment of the burden of drug-resistant TB requires systematic efforts to quantify its magnitude and trend. In approximately half the countries where resistance has been reported, estimates are based on surveys conducted in public sector facilities. However, in locations where a substantial fraction of TB cases seek care with private providers, these surveys may not accurately measure resistance in the entire population.

**Methods:**

We describe a mathematical model to investigate biases associated with sampling only from public sector cases in India, where TB treatment is offered in both public and private sectors. We then propose and demonstrate a weighted estimator as an efficient method for including small numbers of cases from the private sector as a way to recover valid estimates of resistance in the population under study.

**Results:**

We find that public sector surveys rarely provide valid estimates of drug-resistance among new and retreatment cases. Further, the magnitude and direction of the bias are sensitive to many parameters describing the health-seeking behaviours and treatment outcomes of tuberculosis patients, disallowing simple adjustments to recover accurate estimates.

**Conclusions:**

In locations where large numbers of tuberculosis patients are diagnosed and treated by private sector practitioners who are not typically included in drug resistance surveys, targeted surveys for assessing drug resistance are required to validly estimate resistance.

## Background

*M. tuberculosis *resistant to the most potent available antibiotics threatens the success of global tuberculosis (TB) control strategies that rely on standard combinations of these drugs [1]. Although resistance to even a single first-line agent can reduce the probability of successful treatment outcome,[2] the most worrisome disease is resistant to both isoniazid and rifampin (multidrug-resistant; MDR). At least 50% of MDR TB patients treated with standard first-line therapy will fail treatment and suffer from relapse, chronic disease, or die [3]. Furthermore, the detection of extensively drug resistant tuberculosis (XDR),[4,5] defined as MDR with additional resistance to at least a fluoroquinolone and a second-line injectable antibiotic, heightens concern that increasingly resistant forms of tuberculosis will undermine the effectiveness of the current arsenal of treatment [6].

Determining the burden of MDR TB requires systematic efforts to assess the magnitude and trend of drug-resistant disease. Since 1994, under the leadership of the World Health Organization and the International Union Against Tuberculosis and Lung Diseases (WHO-IUATLD), four Anti-Tuberculosis Resistance in the World reports have been published [1,7]. These reports include data from countries and regions that conduct either continuous surveillance or periodic surveys for drug resistant disease. The WHO-IUATLD provides guidance for the design of such surveys; the principle requirements are that they 1) include a representative sample of TB cases from the area under study; 2) differentiate between TB cases that have and have not been previously exposed to anti-TB antibiotics; and 3) use standard methods for determining drug resistance and utilize approved reference laboratories for quality assurance [8].

In some settings, obtaining representative samples of specimens from new and retreatment patients is a challenge. While drug resistance surveys are usually coordinated in the public sector by National Tuberculosis Programs (NTPs), in areas where a substantial fraction of patients are treated by private practitioners, many patients are neither observed in the public sector nor notified to public authorities. Consequently, surveys conducted exclusively on public sector cases may not reflect the local burden of resistance.

Here, we describe a simple mathematical model to investigate potential biases associated with sampling only from public sector cases in India. We use India as our motivating example to examine such biases for two reasons. First, India has the highest burden of TB patients in the world and most patients initially seek care through private practitioners [9,10]. Second, previous work in India provides information on differences in diagnostic and treatment practices in the public and private sector (each of which are likely to affect the acquisition of drug resistance) and behavioral data suggesting how TB patients move between the private and public sectors [11]. These data are have not been measured and are not generally available for other countries with both a high burden of TB and a sizable private sector in which diagnosis and treatment of TB is common. We present general conditions under which surveys conducted in the public sector provide valid estimates of drug resistance among new, retreatment, and combined cases and suggest methods to test whether these conditions are met. Finally, when exclusion of private sector cases from surveys results in biased estimates of the local burden of resistance, we describe an efficient method for including data from private sector samples to produce valid estimates of resistance.

### Private sector providers and drug-resistant tuberculosis in India

India has the largest estimated number of incident (almost 2 million new cases per year) and prevalent TB cases (almost 3.5 million existing cases), representing more than 20% of the total worldwide burden of TB [12]. India has invested heavily in extending free access to high-quality, standardized approaches for diagnosis and directly observed treatment of TB through public sector facilities and since 2006 has offered such services in all districts of the country. Still, surveys indicate that between 50%-90% of Indian patients first seek care through a private practitioner; this preference for care delivered in the private sector exists among patients with major and minor illnesses, patients residing in rural and urban areas, and patients from rich and poor households [9,10,13,14]. Patients may first seek care with for-profit providers despite the fees associated with such services because private sector care is viewed as more accessible and for-profit providers are perceived as more empathic and better able to dispense high quality drugs [11].

While systematic steps toward improving the quality of TB diagnosis and treatment in the private sector through the WHO Public-Private Mix DOTS model (PPM-DOTS) in India appear promising,[15,16] most care provided by private practitioners outside of PPM-DOTS programs remains sub-optimal. Specifically, private providers outside of PPM-DOTS do not routinely use sputum smear microscopy to diagnose TB, are more likely to prescribe non-standard regimens of antibiotics, and do not directly observe treatment to ensure adherence [13,17,18]. TB patients treated by these private sector providers are thus less likely to complete treatment and, if they survive and are not lost from the health care system entirely, will eventually present for retreatment in either the public or the private sector [19]. TB patients treated with inappropriate drugs or for whom adherence is interrupted also have a higher probability of acquiring drug resistance while on treatment.

Based on eight surveys conducted between 1995 and 2006 that included a total of 3562 patients, 2.8% of new TB patients were estimated to be infected with MDR strains [1,7]. Only one survey has provided data necessary to estimate the proportion of incident retreatment cases that are drug-resistant; in this 2006 survey of 1047 retreatment cases, 17.4% of cases were infected with MDR strains. Because these drug-resistance surveys are conducted in public sector facilities, it is not clear whether these surveys can be used to infer the level of drug resistance among all TB patients in India. Furthermore, because the risks of successful treatment outcome and acquired drug resistance differ between public and private patients and because patients failing therapy in one sector may switch to the other for retreatment, understanding the relationship between sector-specific treatment outcomes and the patterns in which patients navigate the healthcare system are important for determining biases associated with drug resistance surveys conducted only in the public sector.

## Methods

### Model details and equations

**Figure 1:**
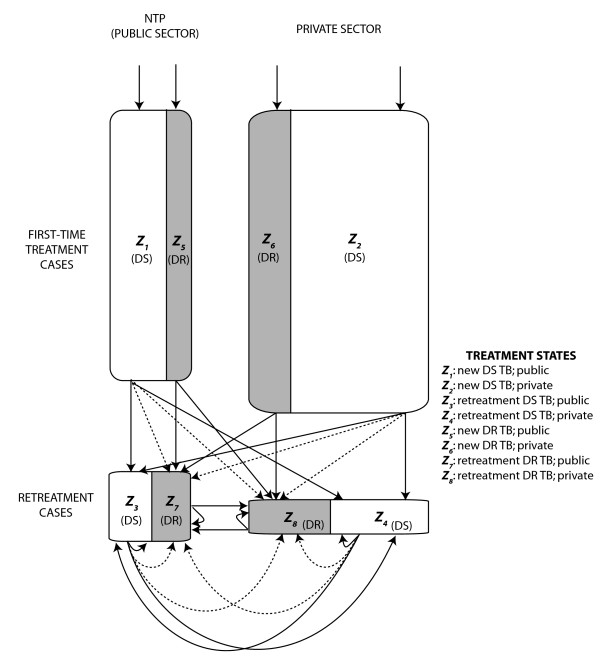
**Model structure**. The model simultaneously distinguishes new TB cases (*Z*_*1*_, *Z*_*2*_, *Z*_*5*_, *Z*_*6*_) from retreatment TB cases (*Z*_*3*_, *Z*_*4*_, *Z*_*7*_, *Z*_*8*_) and characterizes the sector of presentation (public = *Z*_*odd*_; private = *Z*_*even*_) and the drug resistance phenotype (sensitive = *Z*_*≤4*_; resistant = *Z*_*>4*_). Shaded areas represent classes of drug resistant disease while white areas represent classes of drug sensitive disease. The equations that describe transitions between states are provided in the text. The dotted arrows represent the acquisition of drug resistance from one treatment episode to the next.

We developed a simple conceptual mathematical framework to evaluate the conditions under which surveys of drug-resistant TB that are conducted in the public sector may produce valid estimates of the level of drug-resistant disease in the entire population. We adopt a difference equation model in which the population of prevalent TB cases is divided into eight compartments: new cases of drug-sensitive TB in the public sector (*Z*_1_), new cases of drug-resistant disease in the public sector (*Z*_5_), retreatment cases of drug-sensitive disease in the public sector (*Z*_3_), retreatment cases of drug-resistant TB in the public sector (*Z*_7_), plus four corresponding groups of TB patients which have presented for diagnosis and treatment in the private sector (*Z*_2_, *Z*_6_, *Z*_4 _and *Z*_8_) (Figure 1). Entrance into and transitions between compartments are governed by the following set of difference equations and parameters provided in Table 1. In this model, each time step (*t*) represents an entire treatment generation; that is, the interval between when a case is diagnosed and initiates treatment until that case ultimately resolves as cured, failed, lost to follow-up or died.

**Table 1:** Model input parameters and outputs

Parameter	Meaning	Notes on values; (allowable ranges)
**Inputs**		

Λ	TB incidence rate (new cases)	Fixed incidence (value irrelevant)

*k*_*FN*_	Fraction of new cases that go to the public sector	(0-1)

*k*_*RN*_	Fraction of retreatment cases that go to the public sector	(0-1)

*y*_t_	Fraction of new cases that are sensitive	1-*qC *Where *q *is the relative transmissibility of resistant compared with sensitive strains (range of *q *from 0-1; assumes no resistant "superbugs") and *C *(range of *C *from 0-1) is the proportion of all incident cases with MDR

*x*_*S*_	Fraction of new sensitive cases that go to the public sector	(0-1)

*x*_*R*_	Fraction of new resistant cases that go to the public sector	(0-1)

*f*_*R*_, *f*_*S*_ *f*_*•N*_, *f*_*•P*_	Fraction failing treatment among drug resistant and sensitive Fraction failing treatment in public and private sectors	*f*_*S *_≤ *f*_*R *_Failure more likely for resistant and *f*_*•N *_≤ *f*_*•P *_Failure as or more likely in private sector

*a*_*N*_,*a*_*P*_	Fraction of TB cases in the public and private sectors who acquire resistance	(0-1) *a*_*N *_≤ *a*_*P *_Acquired resistance as or more likely in private sector

*l*	Fraction that are lost to follow-up or die	(0-1)

*r*_*N*_	Fraction in the public sector that return to the public sector for next treatment episode	Retreatment cases more likely to present in public sector

*r*_*P*_	Fraction in the private sector that return to the private sector for next treatment episode	Retreatment cases more likely to present in public sector

**Outputs**		

_*A*_	Proportion of new cases with resistant TB	

*A*_*N*_	Proportion of new cases in public sector with resistant TB	

*A*_*P*_	Proportion of new cases in private sector with resistant TB	

_*B*_	Proportion of retreatment cases with resistant TB	

*B*_*N*_	Proportion of retreatment cases in public sector with resistant TB	

*B*_*P*_	Proportion of retreatment cases in private sector with resistant TB	

*C*	Proportion of all cases with resistant TB	

*C*_*N*_	Proportion of all cases in public sector with resistant TB	

*C*_*P*_	Proportion of all cases in private sector with resistant TB	

We model a constant incidence of tuberculosis (Λ) and assume that the fraction of incident new cases that is drug-resistant (1-*y*_*t*_) reflects the relative frequency of drug-resistant disease in the most recent treatment generation and the relative transmissibility of drug-resistant compared with drug-sensitive *M. tuberculosis*. New tuberculosis cases that are not cured and do not die or get lost to follow-up, appear in the next time step (*t*+1) as retreatment cases. Previously treated cases can be repeatedly re-treated in either the same health sector or switch to the other health sector. Consistent with data from India [9-11], we specify that the majority of cases initially present for treatment in the private sector (*y_t _*(1-2*x_s_*) + (1-*y_t_*) (1-2*x_R_*) > 0), but then move preferentially to the public sector for retreatment (*r_p _*≤1/2 <*r_N_*).

For simplicity, we model only two disease phenotypes: pan-sensitive TB and MDR TB. We model the acquisition of the MDR phenotype as occurring during a single course of treatment (a fraction *a*_*N *_or *a*_*P *_acquire resistance). While this does not realistically reflect the complexity of sequential acquisition of drug resistance, we gain qualitative insight into the effect of treatment and health seeking behavior on the frequency of drug resistance. Additionally, we ignore the possibility that those in retreatment categories may have been re-infected. We consider only the effects of standard first-line drug regimens; as such, the proportion of drug-sensitive cases that are cured exceeds that of drug-resistant cases in both public and private sectors. In all cases, we assume that a higher proportion of both drug-sensitive and drug-resistant cases are *successfully *treated in the public sector than in the private sector.

The fraction of new cases that is resistant reflects both the relative number of existing infectious cases of drug-resistant and drug-sensitive TB and the relative transmissibility of drug-resistant compared with drug-sensitive *M. tuberculosis*. The fraction of retreatment cases that is resistant reflects the past incidence of drug-resistance among new cases, the risk of acquired resistance among individuals initially infected with drug-sensitive strains, and the relative effectiveness of treatment for those with drug-resistant compared to drug-sensitive disease. To evaluate conditions under which surveys conducted in the public sector are biased, we report percent bias comparing measures of the proportion of disease that is resistant among new and retreatment cases in the public sector to these same measures in the counterfactual scenario where cases from both the public and private sector are sampled.

### Resistance among new cases

Let *C *represent the overall prevalence of MDR TB in all new cases, and *A_N _*and *A_p _*represent the prevalence of MDR TB in new cases in the public and private sectors, respectively. We deconstruct *A *= *k*_*FN*_*A*_*N *_+ (1-*k*_*FN*_)*A*_*p*_, where *k_FN _*is the proportion of new cases that present at the public sector. What this shows is that in order to know or estimate *A *we need knowledge of both *A*_*N *_and *A*_*P*__, _that is knowledge that can only be assumed, or obtained from measuring both sectors. This equation can be rewritten as

It immediately follows that *A_N _*is equal to *A*, and therefore an unbiased estimator of *A*_*N *_based on a random sample from the public sector will unbiasedly estimate *A*, if and only if *k_FN _*= 1 and, or *A_N _*= *A_p_*. First, all new cases go to the public sector (i.e. *k_FN _*= 1) if and only if all new sensitive and all new drug-resistant cases go to the public sector (i.e. *x_S _*= *x_R _*= 1). Second, if all new cases do not go to the public sector (i.e. *x_S _*≠ 1 or *x_R _*≠ 1), then *A_N _*must equal *A_p _*for *A*_*N *_to also equal *A*. But *A_N _*= *A_P _*requires that the fraction of new sensitive cases that go to the public sector is the same as the fraction of new resistant cases that go to the public sector (i.e. *x_S _*= *x_R_*), otherwise, if *x_S _*>*x_R_*, then *A_N _*<*A_P _*which leads to *A_N _*<*A*; and conversely, if *x_S _*<*x_R_*, then *A_N _*>*A_P_*, which leads to *A_N _*<*A*.

### Resistance among retreatment cases

Let *B *represent the overall prevalence of MDR TB in all retreatment cases, and *B_N _*and *B_P _*represent the prevalence of MDR TB in retreatment cases in the public and private sectors respectively. We deconstruct *B *= *k *_*RN*_*B*_*N *_+ (1-*k_RN_*)*B_P_*, where *k_RN _*is the proportion of retreatment cases that present at the public sector. This can be rewritten as

It immediately follows that *B_N _*equals *B*, and an unbiased estimator of *B*_*N *_based on a sample from the public sector unbiasedly estimates *B*, if and only if *k_RN _*= 1 and, or *B_N _*= *B_P_*. Relating this to the parameters in the mathematical model, *k_RN _*= 1 requires all individuals to attend public facilities for retreatment regardless of where previously treated (i.e. *r*_*N *_= 1 - *r*_*P *_= 1). In general, if an equal fraction of individuals treated in the public sector and individuals treated in the private sector seek retreatment in the public sector (0 ≤ *r*_*N *_= 1 -*r*_*P *_≤ 1), then it follows immediately that *B*_*N *_= *B*_*P*_.

### Resistance among combined cases

Let *C *represent the prevalence of MDR TB in all cases. We deconstruct *C *in terms of prevalence of drug resistance in new and retreatment cases, *C *= *k*_*N*_*A *+ (1 - *k*_*N*_)*B*, where *k*_*N *_is the proportion of all cases that are new. Presuming that we unbiasedly estimate *A *and *B*, these estimates must be weighted by the proportion of new and retreatment cases in order to unbiasedly estimate *C*.

### Investigating the role of small targeted surveys

In the event that the conditions required for public sectors surveys to return unbiased estimates of resistance among new or retreatment cases are unlikely to be met, we recommend a weighted estimator to combine data from public and private sectors to unbiasedly estimate MDR TB prevalence in new and retreatment cases. We assume that the relative burden of cases for public and private sector are known. Presuming that the MDR TB surveys are routine activities in the public sector, the data from these surveys can be augmented with data from targeted surveys in the private sector to efficiently provide unbiased estimates of the MDR TB prevalence (Case 1) [20]. In Case 1, *n*_*N*_, the number of samples collected in the public sector, is fixed by the routine activity, and *n*_*P*_, the number of samples collected from the private sector, is determined by either resources or desired level of accuracy. For example, suppose on a routine basis, 200 samples are tested in the public sector (for each type of case) to estimate the prevalence of MDR TB. To obtain an unbiased estimate of the fraction of all cases with resistance, we can sample an additional 50 individuals in the private sector. The choice of 50 additional samples is arbitrary and was chosen only for illustrative purposes. When surveys are not routine in the public sector, we propose an activity that divides the sample between the two sectors (Case 2). In Case 2, the total sample size, *n*, will be determined by resource availability and will then be divided between the public and private sector, so that *n *= *n*_*N *_+ *n*_*P*_. For example, if resources only allow testing of 50 samples, we may divide the samples evenly between the public and private sectors.

## Results

### Resistance among new cases

**Figure 2:**
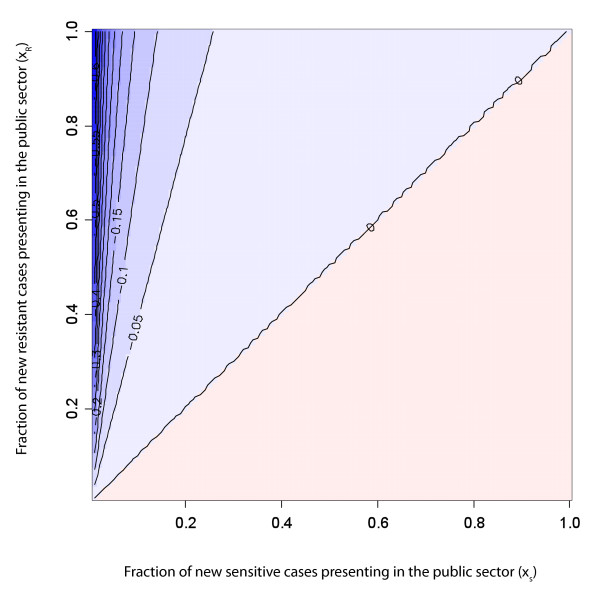
**Bias in new cases**. Percent bias in new cases as a function of the fraction of sensitive cases presenting to the public sector (*x*_*S*_) and the fraction of resistant cases presenting to the public sector (*x*_*R*_). Blue represents parameter space in which public sector surveys overestimate total resistance and red represents parameter space in which public sector surveys underestimate total resistance; more saturated colors indicate greater bias. The values on the lines indicate percent bias. Results reported for models at equilibrium with *a*_*N *_= 0.1; *a*_*P *_= 0.1; *f*_*SN *_= 0.1; *f*_*SP *_= 0.1; *f*_*RN *_= 0.25; *f*_*RP *_= 0.25; *l *= 0.2; *q *= 0.6; *r*_*N *_= 0.85; *r*_*P *_= 0.5.

Figure 2 shows the bias when estimating the prevalence of MDR TB in new cases solely with information measured on public sector new cases for a range of *x_R _*and *x_S_*, for a system at equilibrium. As shown in the methods section, in order to unbiasedly estimate overall prevalence in new cases with data from public clinics, either *i*) all new cases must present in the public sector or *ii*) the proportion of all new drug-sensitive cases that present to the public sector (*x_S_*) must be equal to the proportion of all drug-resistant cases that present to the public sector (*x*_*R*_). Also, if a higher proportion of new resistant cases present to the public clinics than new sensitive cases (*x_R _*>*x_S_*), then the public sector data will over-estimate the prevalence of MDR TB in all new cases. However, if a higher proportion of new sensitive cases present to the public clinics than new resistant cases (*x*_*S *_>*x*_*R*_), then the public sector prevalence of MDR TB in new cases will underestimate the overall prevalence. When the overall fraction of new cases that present in the public sector is low (bottom left-hand corner of the figure), the amount of bias present in surveys of public sector cases is sensitive to even small imbalances between *x_S _*and *x*_*R*_.

### Resistance among retreatment cases

**Figure 3:**
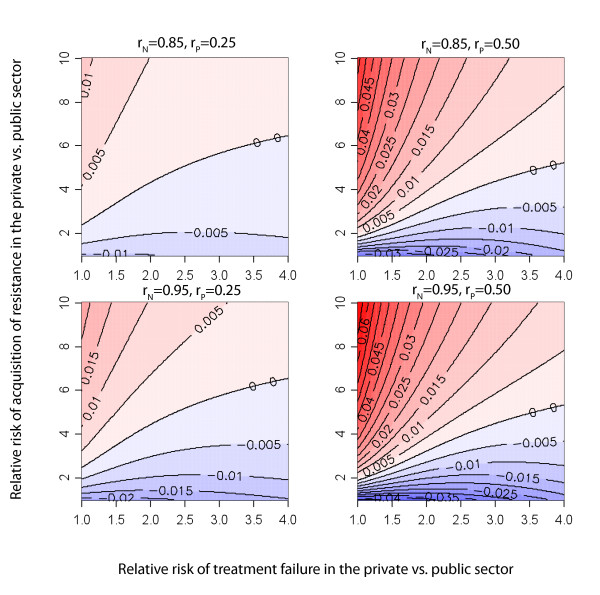
**Bias in retreatment cases**. Percent bias in retreatment cases as a function of the relative risk of acquired drug resistance and relative risk of failure in the private sector. Blue represents parameter space in which public sector surveys overestimate total resistance and red represents parameter space in which public sector surveys underestimate total resistance; more saturated colors indicate greater bias. The values on the lines indicate percent bias. Results present values at equilibrium with *a*_*N *_= 0.1; *a*_*P *_allowed to vary; *f*_*SN *_= 0.1; *f*_*SP *_allowed to vary; *f*_*RN *_= 0.25; *f*_*RP *_allowed to vary; *l *= 0.2; *q *= 0.6; *x*_*R *_= 0.2; *x*_*S *_= 0.2. The panels represent four different scenarios of patient preference for retreatment in public or private sector.

Figure 3 shows the percent bias when estimating the prevalence of MDR TB in retreatment cases solely with information measured on public sector retreatment cases, for a system at equilibrium. As described in the methods section, the only sufficient condition that assures unbiased estimates of the prevalence of MDR TB in retreatment cases with samples obtained only in public settings is for failures from the private and public sectors to present nondifferentially to the public sector for retreatment (*r*_*N *_= 1-*r*_*P*_). If TB patients failing from the public and private sectors have different preferences for where they seek retreatment (*r*_*N *_≠ 1-*r*_*P*_), there will almost certainly be bias in our estimate of drug resistance among retreatment cases. We find that the direction and magnitude of this bias depends on numerous factors including the relative risk (RR) of acquiring resistance in each sector, the RR of treatment failure in each sector, and the sector-specific probability of switching location for retreatment (Figure 3).

For any fixed value of the RR of failure, there is a greater fraction of resistance among retreatment cases in the private sector as the RR of acquired resistance increases within the private sector (trend toward darker red moving up the *y*-axis in Figure 3). Furthermore, this bias is amplified when individuals failing therapy within either sector remain within that sector for retreatment. Additional files provide results where we do not assume equal MDR TB prevalence in new cases (Additional File 1) and when risk of amplified resistance in the public sector exceeds that in the private sector (Additional File 2).

### Resistance among combined cases

**Figure 4:**
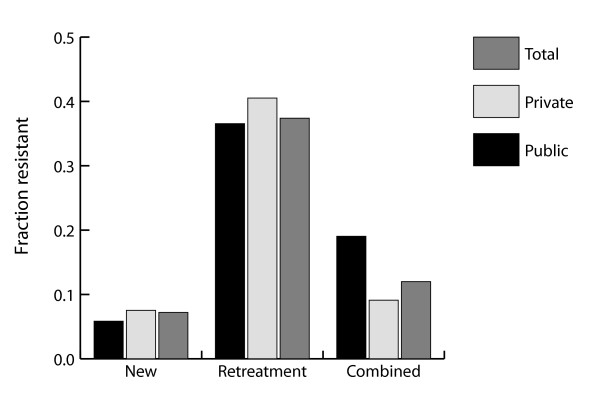
**Bias in combined cases**. Simpson's paradox may occur when the fraction of cases that is drug resistant is only counted among combined cases and not separately among new and retreatment cases. By examining public sector combined cases only we overestimate the total fraction of resistant cases in the population, while among both subcategories (new and retreatment cases) we underestimate the fraction that are resistant. Results present values at equilibrium with *a*_*N *_= 0.1; *a*_*P *_= 0.1; *f*_*SN *_= 0.1; *f*_*SP *_= 0.1; *f*_*RN *_= 0.25; *f*_*RP *_= 0.25; *l *= 0.2; *q *= 0.6; *r*_*N *_= 0.85; *r*_*P *_= 0.5; *x*_*R *_= 0.16; *x*_*S *_= 0.2

While current guidelines strongly recommend reporting resistance among new and retreatment cases separately, some surveys have reported resistance only among new and retreatment cases combined. For example, in a 1995 survey conducted in the Delhi State of India, a survey of 2240 incident TB cases found 13.3% of these cases were MDR, but did not identify the fraction resistant within new and retreatment categories. Figure 4 illustrates a potential problem with the interpretation of resistance among combined cases: while it is possible that resistance among both new and retreatment cases is actually *underestimated *when observing cases in only the public sector, the observed resistance among combined cases may actually be an *overestimate *of resistance in the population. This is the consequence of retreatment cases (whom we know to be at increased risk of resistance) presenting preferentially in the public sector where these surveys are completed and provides an example of Simpson's paradox [21,22]. This situation arises because the new and retreatment cases are not given the appropriate weights when only combined cases are reported.

### Use of small targeted surveys to eliminate bias

We consider two scenarios to illustrate the potential applications of this approach. First, in locations where there is ongoing surveillance for drug resistance in the public sector, we propose a simple extension to estimate prevalence of drug resistance in the private sector (Case 1). Second, in areas where public sectors surveys are not routinely conducted, we propose an efficient sampling strategy that returns unbiased estimates of resistance for a fixed sample size (Case 2).

For new cases, the unbiased estimator of MDR prevalence is *Â *= *k*_*FN *_*Â*_*N *_+ (1-*k*_*FN*_) *Â*_*P *_and the variance for this estimator is

A similar estimator and variance can be constructed for retreatment cases. The variances of the estimators differ between the two scenarios based on the sample sizes determination.

**Figure 5:**
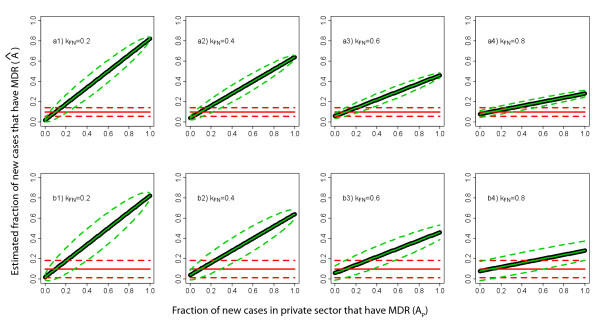
**Comparing estimated resistance in new cases in surveys from public sector only and surveys from public and private sectors**. Estimates (solid colored lines) and variances (dotted lines) for new cases based on public sector only data (red) and public and private sector data (green), assuming different fractions (*k*_FN_) of new cases seek care in the public sector, of which 10% have MDR TB. The thick black line represents the actual underlying proportion of drug resistance among new cases. The top panels (a1-a4) shows Case 1 (described in the text) where 50 additional samples from the private sector are used to create an improved estimate, the bottom panels (b1-b4) shows Case 2 where the total sample is evenly split between public and private sectors.

The impact of using these estimators to correct for biases depends on both the sector-specific burden of disease and prevalence of drug resistance. Figures 5a1 and 5b1 depict a plausible scenario for new cases, where we presume the fraction of cases with drug resistance in the public sector is 10%, but only 20% of patients initially receive care in the public sector. Except the special case when there is also 10% resistance among new cases in the private sector, the public-sector-only estimates are biased. The bias increases sharply as resistance in the private sector diverges from that in the public sector. For Case 1, including an additional 50 samples from the private sector will eliminate bias in the estimator, with little compromise in variance. For Case 2, splitting the sample between the public and private sectors will also eliminate bias, though the accuracy of the estimators will decrease. The remaining of the panels of Figure 5 show scenarios with increasing fractions of new patients presenting in the public sector.

**Figure 6:**
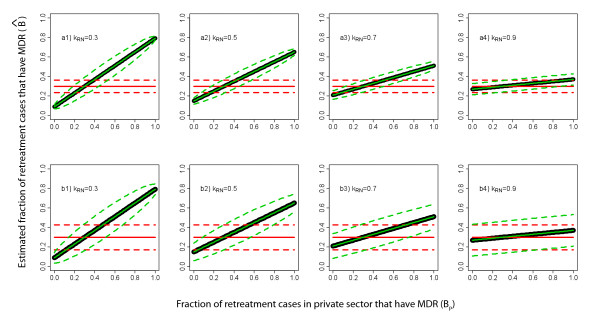
**Comparing estimated resistance in retreatment cases in surveys from public sector only and surveys from public and private sectors**. Estimates (solid colored lines) and variances (dotted lines) for retreatment cases based on public sector data only (red) and public and private sector data (green), assuming different fractions (*k*_RN_) of retreatment cases seek care in the public sector, of which 30% have MDR TB. The thick black line represents the actual underlying proportion of drug resistance among new cases. The top panels (a1-a4) shows Case 1 (described in the text) where 50 additional samples from the private sector are used to create an improved estimate, the bottom panels (b1-b4) shows Case 2 where the total sample is evenly split between public and private sectors.

A similar situation applies for retreatment cases, where, for illustration, we assume 30% of MDR TB in the public sector and 70% of individuals seeking care in the public sector for retreatment (Figure 6a3 and 6b3). Again, sampling in the private sector eliminates bias from the estimator. The variance of this unbiased estimator is again smallest when adding resources to ongoing activities (Case 1), instead of splitting resources between the two sectors (Case 2). However, we note that for retreatment cases in India, the bias is likely to be smaller than for new cases since a smaller proportion of retreatment cases seek care in the private sector. The remaining of the panels of Figure 6 show scenarios with different fractions of retreatment patients presenting in the public sector.

## Discussion

Accurate estimates of the burden of MDR tuberculosis are required to mobilize appropriate resources to combat this threat to global tuberculosis control. In locations where patients can access care in various settings, patterns of care-seeking behavior may be related to drug-resistance. For example, there may be socio-economic factors that are associated with both infection with drug-resistant strains and preference for private sector care or, in areas where second-line treatment for MDR-TB is only offered in one sector, patients with suspected MDR may preferentially move to that sector for treatment. There are few data to suggest how care-seeking behavior may be associated with drug-resistance within any particular location, and, even if these associations were well-measured it would be difficult to generalize these findings to new settings where resistance and treatment may have a very different association.

Our model presents a simplified picture of the mechanisms by which drug resistance appears and spreads within populations and provides insight into the conditions when surveys of drug-resistant disease will be biased in settings where treatment is offered by both private and public practitioners. We find that stringent conditions must be met to assure that resistance among new and retreatment cases is unbiasedly estimated with public sector surveys (Figures 2 and 3). Furthermore, especially when estimating resistance among retreatment cases, it is difficult to predict either the *magnitude *or the *direction *of this bias. As the data necessary for trying to infer direction and magnitude of bias is rarely known (and probably never known with sufficient certainty), we propose a simple method for using weighted estimators that requires additional information from the private sector to be combined with estimates from public sector surveys to efficiently and validly estimate resistance in the entire population. In countries with ongoing or routine MDR TB surveys in the public sector, targeted surveys in the private sector provide an efficient mechanism to remove bias. However, even in locations without existing surveillance in the public sector, the stratified sampling estimator can be used to initiate public/private sector surveys (Figures 5 and 6).

In order to obtain this unbiased estimator of MDR TB in the new and retreatment cases using the stratified sampling estimator, we assumed that the burden of disease for each sector is known, in order to directly calculate *k*_*FN *_and *k*_*RN *_(the fraction of new and retreatment cases, respectively, that go to the public sector). Knowledge of the burden of TB by sector also would allow for proportionate division of our sample under fixed resource assumptions (Case 2) in order to minimize the variance of the estimator. In practical terms, this will require national TB programs to strengthen relations with private sector and create mechanisms for reliable notification (or estimation) of new and retreatment disease in both sectors; this remains a immense challenge, especially in areas with many types of private providers and hidden populations of patients receiving informal care. However, as has been noted previously [15,16], an additional benefit of increasing communication and contact with private providers promises to improve the standard of care for patients being treated outside of the public system.

Our conclusions are based on a crude model for the health seeking behavior of new and retreatment TB cases. Since we were focused on understanding the relationship between health seeking behavior and treatment outcomes of patients on the performance of tuberculosis drug resistance surveys, the model does not include details of the complex natural history of tuberculosis. For example, we do not include age-specific differences in the rates of progression to disease or the risks of reinfection. We also assume a fixed time-step over which cases present for retreatment and that this time-step does not depend on drug resistance phenotype. Omitting these details allows us to gain insight into the potential biases associated with surveys limited to the subset of patients attended to in the public sector, but limits our ability to assess the dynamic behavior of this system. Accordingly, we focus our conclusions on the system in equilibrium. We note that when levels of drug resistance are rising or falling, the potential biases associated with public-sector only surveys will be even more unpredictable, further supporting our recommendation that small surveys in the private sector will be important for understanding the true burden of resistance in locations where patients seek care outside the public system.

## Conclusions

Recent recommendations of the WHO and IUATLD recognize the need to assess drug resistance within the private sector in countries where care is offered in settings where surveys are not easily implemented,[23] however these recommendations currently emphasize the need to first expand and improve surveys within the public sector. While we agree that public sector surveys remain a central activity, we suggest that even small additional surveys targeted to the private sector would substantially improve validity of these surveys. In the absence of adequate scale-up of second-line treatment programs for MDR care in the public sector, we expect that suspected MDR cases will increasingly seek care with private providers offering non-standardized care; this would worsen bias and increase the likelihood that resistance among retreatment cases would be underestimated if only measured within the public sector.

## Competing interests

The authors declare that they have no competing interests.

## Authors' contributions

TC and BH conceived of the study, developed the model, and wrote the first draft of the manuscript. MP participated in the design of the study and revisions of the manuscript. All authors read and approved the final manuscript.

## Pre-publication history

The pre-publication history for this paper can be accessed here:

http://www.biomedcentral.com/1471-2458/10/355/prepub

## Supplementary Material

Additional file 1 **Bias in retreatment cases when there is not necessarily equal prevalence of MDR among new cases presenting to public and private sectors**. Percent bias in retreatment cases as a function of the relative risk of acquired drug resistance and relative risk of failure in the private sector. Blue represents parameter space in which public sector surveys overestimate total resistance and red represents parameter space in which public sector surveys underestimate total resistance; more saturated colors indicate greater bias. The values on the lines indicate percent bias. Results present values at equilibrium with *a *= 0.1; *f*_*S *_= 0.1; *f*_*R *_= 0.25; *l *= 0.2; *q *= 0.6; *r*_*N *_= 0.85; *r*_*P *_= 0.5. The panels represent four alternative possibilities for the likelihood and preference for new MDR cases to present in the public or private sector. This figure should be compared with Figure 3 of the main text. Click here for file

Additional file 2 **Bias in retreatment cases under the assumption that the probability of acquisition of resistance is lower in the private sector**. Percent bias in retreatment cases as a function of the relative risk of acquired drug resistance and relative risk of failure in the private sector when acquired resistance is more common for patients treated in the public sector. The values on the lines indicate percent bias. Results present values at equilibrium with *a*_*N *_= 0.1; *a*_*P *_allowed to vary; *f*_*SN *_= 0.1; *f*_*SP *_allowed to vary; *f*_*RN *_= 0.25; *f*_*RP *_allowed to vary; *l *= 0.2; *q *= 0.6; *x*_*R *_= 0.2; *x*_*S *_= 0.2. The panels represent four different scenarios of patient preference for retreatment in public or private sector. In each of these scenarios, public sector surveys overestimate total resistance. This figure should be compared with Figure 3 of the main text. Click here for file
