# Supplementary material for: Estimating the magnitude and direction of bias in tuberculosis drug resistance surveys conducted only in the public sector: a simulation study
Source: BMC Public Health. 2010 Jun 21;10:355. doi: 10.1186/1471-2458-10-355 (PMC2898828; doi:10.1186/1471-2458-10-355)
Supplement: Additional file 1 — Bias in retreatment cases when there is not necessarily equal prevalence of MDR among new cases presenting to public and private sectors. Percent bias in retreatment cases as a function of the relative risk of acquired drug resistance and relative risk of failure in the private sector. Blue represents parameter space in which public sector surveys overestimate total resistance and red represents parameter space in which public sector surveys underestimate total resistance; more saturated colors indicate greater bias. The values on the lines indicate percent bias. Results present values at equilibrium with a = 0.1; fS = 0.1; fR = 0.25; l = 0.2; q = 0.6; rN = 0.85; rP = 0.5. The panels represent four alternative possibilities for the likelihood and preference for new MDR cases to present in the public or private sector. This figure should be compared with Figure 3 of the main text. [file 1471-2458-10-355-S1.PDF]

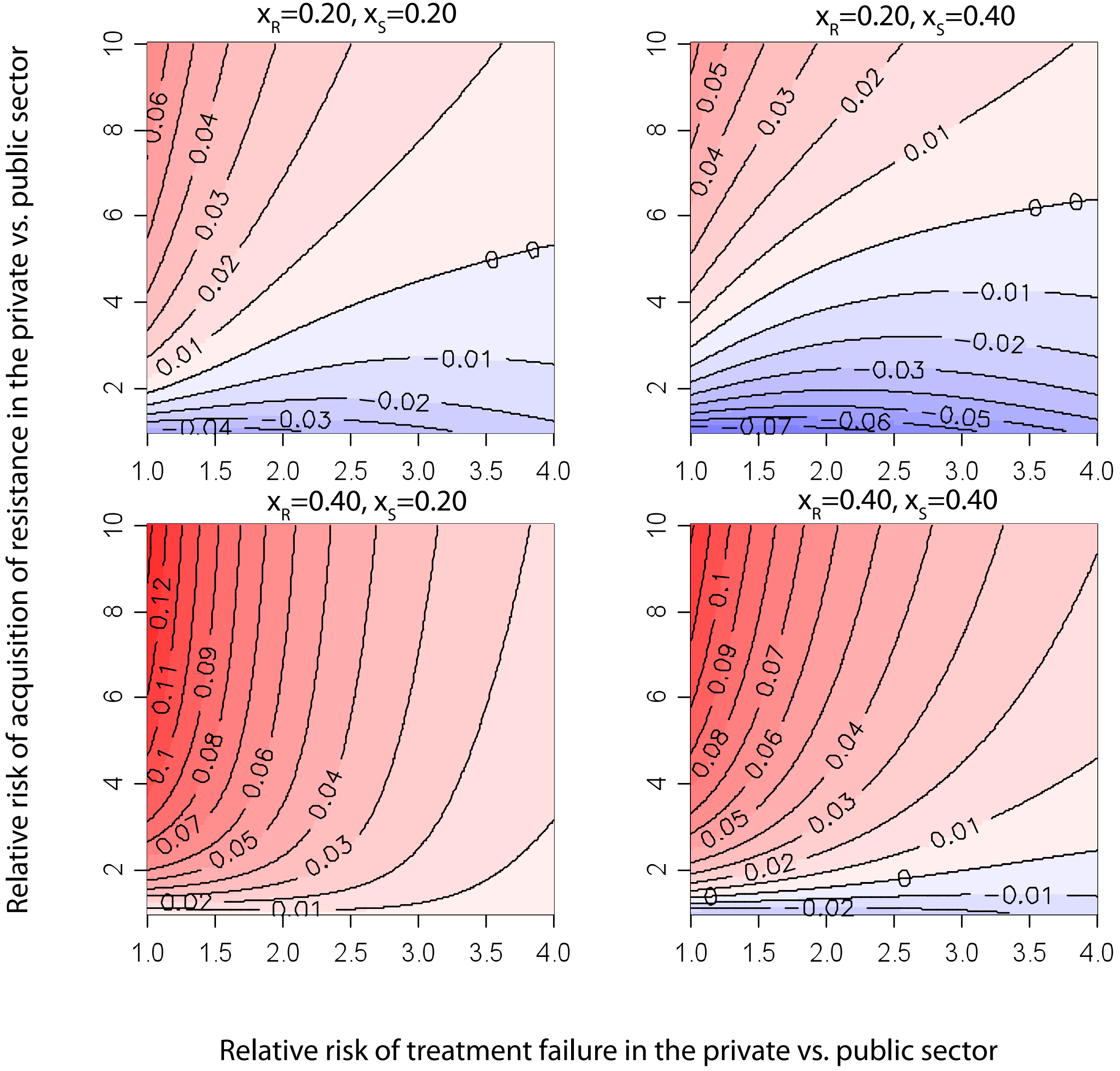

Relative risk of acquisition of resistance in the private vs. public sector
Relative risk of treatment failure in the private vs. public sector
x_R=0.20, x_S=0.20
x_R=0.20, x_S=0.40
x_R=0.40, x_S=0.20
x_R=0.40, x_S=0.40
