# Supplementary material for: Estimating the magnitude and direction of bias in tuberculosis drug resistance surveys conducted only in the public sector: a simulation study
Source: BMC Public Health. 2010 Jun 21;10:355. doi: 10.1186/1471-2458-10-355 (PMC2898828; doi:10.1186/1471-2458-10-355)
Supplement: Additional file 2 — Bias in retreatment cases under the assumption that the probability of acquisition of resistance is lower in the private sector. Percent bias in retreatment cases as a function of the relative risk of acquired drug resistance and relative risk of failure in the private sector when acquired resistance is more common for patients treated in the public sector. The values on the lines indicate percent bias. Results present values at equilibrium with aN = 0.1; aP allowed to vary; fSN = 0.1; fSP allowed to vary; fRN = 0.25; fRP allowed to vary; l = 0.2; q = 0.6; xR = 0.2; xS = 0.2. The panels represent four different scenarios of patient preference for retreatment in public or private sector. In each of these scenarios, public sector surveys overestimate total resistance. This figure should be compared with Figure 3 of the main text. [file 1471-2458-10-355-S2.PDF]

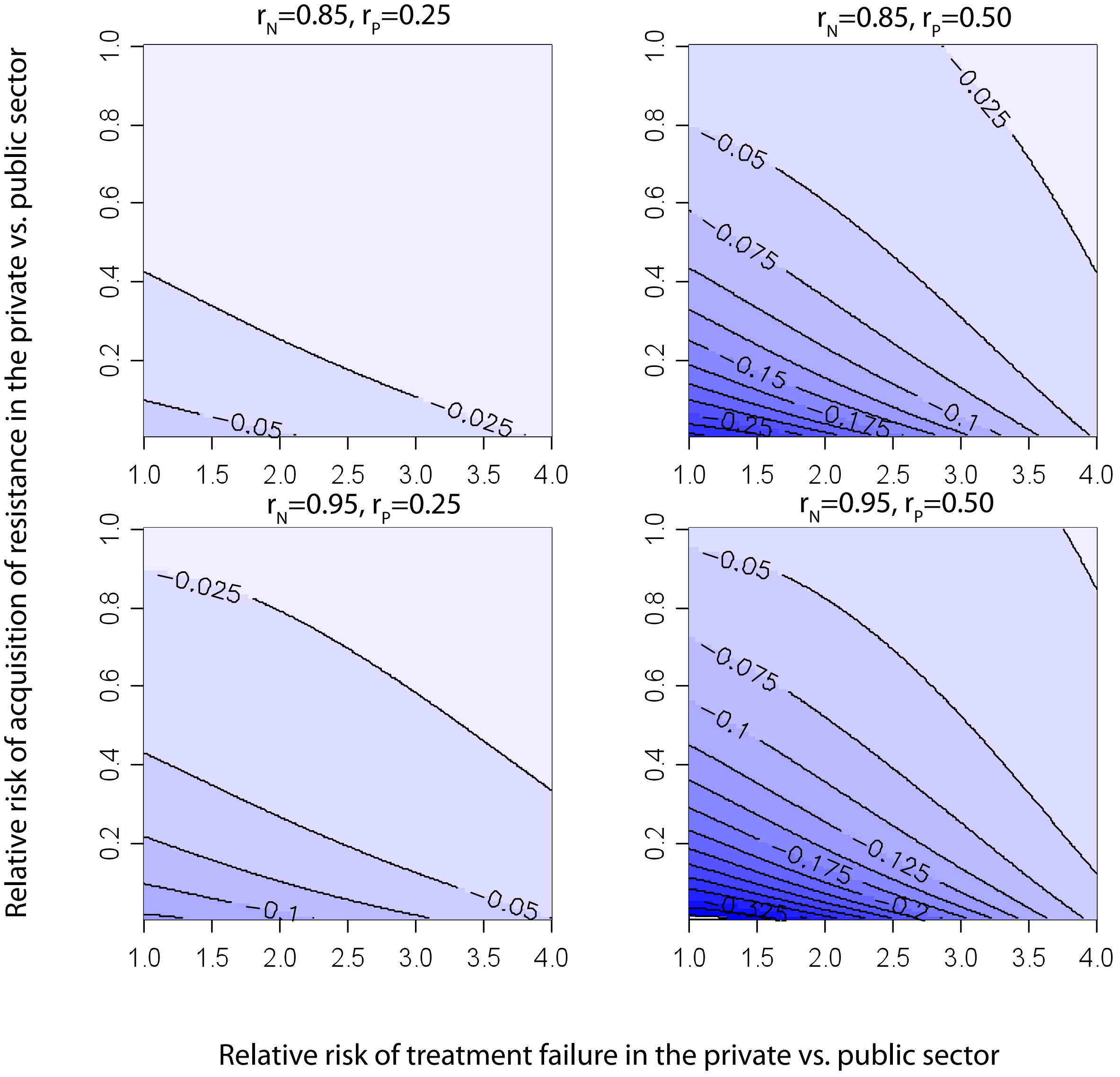

r_N=0.85, r_P=0.25
r_N=0.85, r_P=0.50
r_N=0.95, r_P=0.25
r_N=0.95, r_P=0.50
-0.025
-0.05
-0.075
-0.1
-0.125
-0.15
-0.175
-0.2
-0.25
-0.325
1.0
0.8
0.6
0.4
0.2
1.0 1.5 2.0 2.5 3.0 3.5 4.0
Relative risk of acquisition of resistance in the private vs. public sector
Relative risk of treatment failure in the private vs. public sector
